# Profiling Analysis of Histone Modifications and Gene Expression in Lewis Lung Carcinoma Murine Cells Resistant to Anti-VEGF Treatment

**DOI:** 10.1371/journal.pone.0158214

**Published:** 2016-06-30

**Authors:** Dong Li, Jiejun Shi, Yanhua Du, Kaiming Chen, Zhenping Liu, Bing Li, Jie Li, Fei Tao, Hua Gu, Cizhong Jiang, Jianmin Fang

**Affiliations:** 1 School of Life Sciences and Technology, Tongji University, Shanghai, 200092, China; 2 The Collaborative Innovation Center for Brain Science, Shanghai Key Laboratory of Signaling and Disease Research, Tongji University, Shanghai, 200092, China; Ludwig-Maximilians-Universität München, GERMANY

## Abstract

Tumor cells become resistant after long-term use of anti-VEGF (vascular endothelial growth factor) agents. Our previous study shows that treatment with a VEGF inhibitor (VEGF-Trap) facilitates to develop tumor resistance through regulating angiogenesis-related genes. However, the underlying molecular mechanisms remain elusive. Histone modifications as a key epigenetic factor play a critical role in regulation of gene expression. Here, we explore the potential epigenetic gene regulatory functions of key histone modifications during tumor resistance in a mouse Lewis lung carcinoma (LLC) cell line. We generated high resolution genome-wide maps of key histone modifications in sensitive tumor sample (LLC-NR) and resistant tumor sample (LLC-R) after VEGF-Trap treatment. Profiling analysis of histone modifications shows that histone modification levels are effectively predictive for gene expression. Composition of promoters classified by histone modification state is different between LLC-NR and LLC-R cell lines regardless of CpG content. Histone modification state change between LLC-NR and LLC-R cell lines shows different patterns in CpG-rich and CpG-poor promoters. As a consequence, genes with different level of CpG content whose gene expression level are altered are enriched in distinct functions. Notably, histone modification state change in promoters of angiogenesis-related genes consists with their expression alteration. Taken together, our findings suggest that treatment with anti-VEGF therapy results in extensive histone modification state change in promoters with multiple functions, particularly, biological processes related to angiogenesis, likely contributing to tumor resistance development.

## Introduction

Lung cancer is the most commonly diagnosed human cancer worldwide and accounts for 1.6 million of total new cancer cases and caused about 1.4 million of cancer-related deaths all over the world in 2008 [1]. To date, the majority of lung cancer cases are diagnosed at the advanced stage of the disease, leading to almost impossible curable surgical resection and poor prognosis. In non-small cell lung cancer (NSCLC), complete surgical resection of stage IA disease could lead to a best prognosis with up to 70% five-year survival rate [2]. Beyond surgery, other treatments include chemotherapy, radiotherapy, and palliative care. Target therapy of lung cancer is also applied to patients with advanced lung cancer [3]. Target therapy, such as anti-angiogenic therapy in treatment of NSCLC [4] or anti-EGFR therapy [5] has shown to improve survival of patients, but tumor resistance develops quickly in certain cases leading to cancer recurrence. Thus, better understanding of the underlying molecular mechanism of resistance to these target therapies could improve the treatment efficacy to control the advanced lung cancer and prolong survival of patients. Lung cancer pathogenesis, similar to many other cancers, is initiated by activation of oncogenes or inactivation of tumor suppressor genes, such as mutation of *K-ras* [6] or *p53* [7], *EGFR* amplification [5], COX-2 overexpression [8], or loss of RAR-β expression [9].

Moreover, epigenetic changes, i.e. DNA methylation or histone modification, and aberrant miRNA expression can also inactivate expression of tumor suppressor genes [10–12]. As the key regulator during angiogenesis, VEGF signaling is also regulated by epigenetic factors. For instance, the promoter methylation level of VEGF receptors has impact on their binding efficacy with VEGF in human umbilical endothelial cells (HUVECs) [13]. In another study, Zhang et al. had found correlation between VEGFA stimulation and H3K27ac dynamic at transcriptional regulatory elements which are functionally related to angiogenesis[14]. Besides VEGF, other pivotal factors in angiogenesis are also fine-tuned in epigenetic way, like the placental growth factor (PlGF) and the hypoxia-inducible factor (HIF) [15]. These findings all imply the close bond between epigenetic changes and angiogenesis. Our previous study has found that prolonged use of anti-VEGF agents promoted tumor resistance by upregulating angiogenesis-related genes [16]. However, little is known about the changes of histone modification states in promoters and the role of corresponding epigenetic regulation. Here, we apply a mouse Lewis lung carcinoma (LLC) cell line to profile histone modifications in tumor sensitive sample (LLC-NR cell line) and tumor resistant sample (LLC-R cell line) after anti-VEGF treatment. The results show that histone modification changes consist with gene expression changes. Especially, altered promoter activity of angiogenesis-related genes may help develop resistance to anti-VEGF therapy.

## Materials and Methods

### Cell line and culture

A murine Lewis lung carcinoma cell line LLC was obtained from the Cell Bank of the Chinese Academy of Sciences (Shanghai, China) and cultured in Dulbecco’s modified Eagle's medium (DMEM) medium (Life Technologies, Gaithersburg, MD) supplemented with 10% fetal bovine serum (FBS, Life Technologies) in a humidified incubator with 5% CO_2_ at 37°C. To develop tumor resistance to anti-VEGF therapy, a murine LLC cell line (LLC-R) was developed that was resistant to a potent VEGF inhibitor, VEGF-Trap, through five round of *in vivo* selection by continuous treatment with VEGF-Trap as previously described [16].

### Animal model of lung cancer isografts

Total 20 six- to eight-week-old C57BL/6J female mice were purchased from SLRC Laboratory Animal Co., Ltd. (Shanghai, China) and housed in the specific pathogen-free animal facility in Tongji University (Shanghai, China) for one week before experiments. Mice had *ad libitum* access to filtered tap water in a specific pathogen free animal room under supervision. The facility staff monitored daily animal health care and took care of commercial feed supply, cages and beddings cleaning, animal observation and report, etc. All animal experiments were performed in accordance with the ethical guidelines of the Tongji University Laboratory Animal Care and Use Committee. All surgery was performed under sodium pentobarbital anesthesia, and all efforts were taken to minimize suffering. LLC-R and LLC-NR cells (5 × 10^5^ cells/mouse) were subcutaneously inoculated into the right flanks of C57BL/6J mice (n = 10/group, total 20 mice) to allow tumor isografts to grow to an approximate size of 500 mm^3^ (tumor volume = (length × width × height)/2) and then tumor isograft tissues were dissected and used for further study. Although we applied humane end points in our study, no mice became severely ill or injured prior to the experimental endpoint. All mice were euthanized by intraperitoneal injection of sodium pentobarbital (500 mg/kg, Sigma-Aldrich, St. Louis, MO) in the end of experiments.

### Preparation of nuclei from tumor isografts

Tissue sections of each isograft (up to 150 mg in weight) were prepared for approximately 300 microns in thickness and cross-linked with 1.8% formaldehyde for 15 min at room temperature and formaldehyde was then inactivated by addition of 125 mM glycine. Tissue homogenate were then harvested for extraction of nuclei in 500 μL RNase digestion buffer and digested with 12000 units of micrococcal nuclease at 37°C for 20 min. The samples were held in ice and EDTA solution was added into the samples for a final concentration of 10 mM to stop RNase digestion. The pelleted nuclei were next re-suspended in 500 μL of the sonication buffer and sonicated for three times with 20 s pulses using Qsonica XL-2000 (Qsonica, LLC, Newtown, CT) to break the nuclear membrane. After that, supernatant of the clarified lysates was transferred into a new tube. Total 25 μL of samples were subjected to reversal of the cross-linking and genomic DNA was extracted and separated using gel electrophoresis.

### Chromatin immunoprecipitation-sequencing (ChIP-seq)

Each (3–5 μg) of different histone modification antibodies (i.e. H3K4me3, #ab8580; H3K27me3, #ab6002; H3K36me3, #ab9050; H3K9ac, #ab10812; all from Abcam) were used to immunoprecipitate the above RNase-digested chromatin (10–20 μg) in 400 μL of the ChIP buffer by incubation of these ChIP samples overnight at 4°C with rotation. On the next day, 30 μL of ChIP-Grade Protein G Magnetic Beads (Cell Signaling Technology, Danvers, MA) were added into each sample and incubated for another 2 h. After washed with salt buffers, the immunoprecipitated chromatin was then eluted. The resulted eluted DNA fragments were sequenced using an Illumina HiSeq2000 at BGI-Shenzhen (Beijing Genomics Institute, Shenzhen, China).

### Bioinformatics analysis of ChIP-seq data

ChIP-seq reads were mapped to mm9 assembly of Mus musculus reference genome using Bowtie (v 0.12.7) [17] with up to two mismatches. The genome sequence and gene model annotation were downloaded from UCSC Genome Bioinformatics Site (http://genome.ucsc.edu/). The statistics of total reads and uniquely mapped reads were summarized in S1 Table. Only the uniquely mapped reads were subjected to further analysis. GeneTrack is a bioinformatics software package for storing, querying and visualizing interval oriented data with an algorithm that is suitable for the peaks to have a prior known width [18]. Theoretically, the width of histone modifications is uniform and equal to nucleosomal DNA (about 147 bp). Thus, the aligned ChIP-seq reads were applied for peak calling using GeneTrack software. The gene promoters were defined as 1 kb upstream to 0.5 kb downstream regions relative to the transcription start site (TSS).

### Correlation between gene transcription level and histone modification level in promoters

The gene transcription levels of both drug resistant and non-resistant tumor samples were determined according to our previous study [16]. The values of fragments per kilobase per million fragments mapped (FPKM) calculated by Cufflinks were used to evaluate the transcript abundance.

The histone modification level at promoter was evaluated in two different approaches for comparison purpose. In first approach, raw reads count in each promoter were used to measure the enrichment of each histone modification. The second approach is based on the fitted peak occupancies calculated by GeneTrack. Specifically, the histone modification enrichment is quantified as the sum of fitted peak occupancies whose midpoint located inside the same gene’s promoter region. Then the Spearman correlation coefficients were calculated between transcription levels and histone modification enrichment at promoter of all genes.

### Defined promoter CG content and transcription activity

The promoter classification according to their CpG content was retrieved from previous study [19]. Briefly, HCPs were promoters containing a 500 bp interval, which had a G/C content ≥ 55% and a CpG observed to expected ratio (O/E) ≥ 0.6. However, if gene promoters had less than 500-bp interval with CpG O/E ≥ 0.4, they were classified as LCPs and otherwise the promoters were defined as ICPs.

The transcription activity of each promoter was determined by its histone modification state. The occupancy of histone modification in a promoter was represented by the normalized tag count. The mean value occupancy of H3K4me3, H3K9ac, and H3K27me3 in all promoters was denoted as M(H3K4me3), M(H3K9ac) and M(H3K27me3), respectively. Because both H3K4me3 and H3K9ac are active markers, “active” state should meet the following requirements: occupancy of H3K4me3 ≥ 2 × M(H3K4me3) or occupancy of H3K9ac ≥ 2 × M(H3K9ac) but occupancy of H3K27me3 < M(H3K27me3). In contrast, “repressive” promoters are the ones with occupancy of H3K27me3 ≥ M(H3K27me3) but exclusive of “active” promoters. The “bivalent” promoters are the ones with occupancy of H3K4me3 ≥ 2 × M(H3K4me3) or occupancy of H3K9ac ≥ 2 × M(H3K9ac) and occupancy of H3K27me3 ≥ M(H3K27me3). The rest of promoters are defined as “none” promoters.

### Quantitative PCR

To confirm the peaks identified by ChIP-seq analysis, qPCR was carried out in triplicate by using 20 ng of DNA sample in a 20 μL reaction volume with the SYBR Green master mix (TaKaRa biotechnology, Dalian, China) in a 7500 ABI Prism Sequence Detection System (Applied Biosystems, Foster City, CA) machine. Primers were designed and shown in S2 Table. For each reaction, a mixture contained 2 μL of DNA (Input or IgG-immunoprecipitated sample), 10 μL 2 × SYBR Green master mix, 1 μL (10 μM) of each primer, 0.4 μL Dye II, and 5.6 μL nuclease free H2O. qPCR amplification was set with a pre-amplification cycle at 95°C for 10 min followed by 40 cycles of amplification (10 s at 95°C and 60 s at 60°C). Enrichment in the promoter regions was measured as the percentage of the immune-precipitated samples normalized to the input samples. qPCR data were referred to a biological control sample and presented as means ± SEM. Student’s t test was performed for statistical analysis.

### Data accession

The ChIP-seq data sets have been deposited in Gene Expression Omnibus (GEO) database under accession number GSE70753.

## Results

### Histone modification levels correlate with gene expression

**Fig 1:**
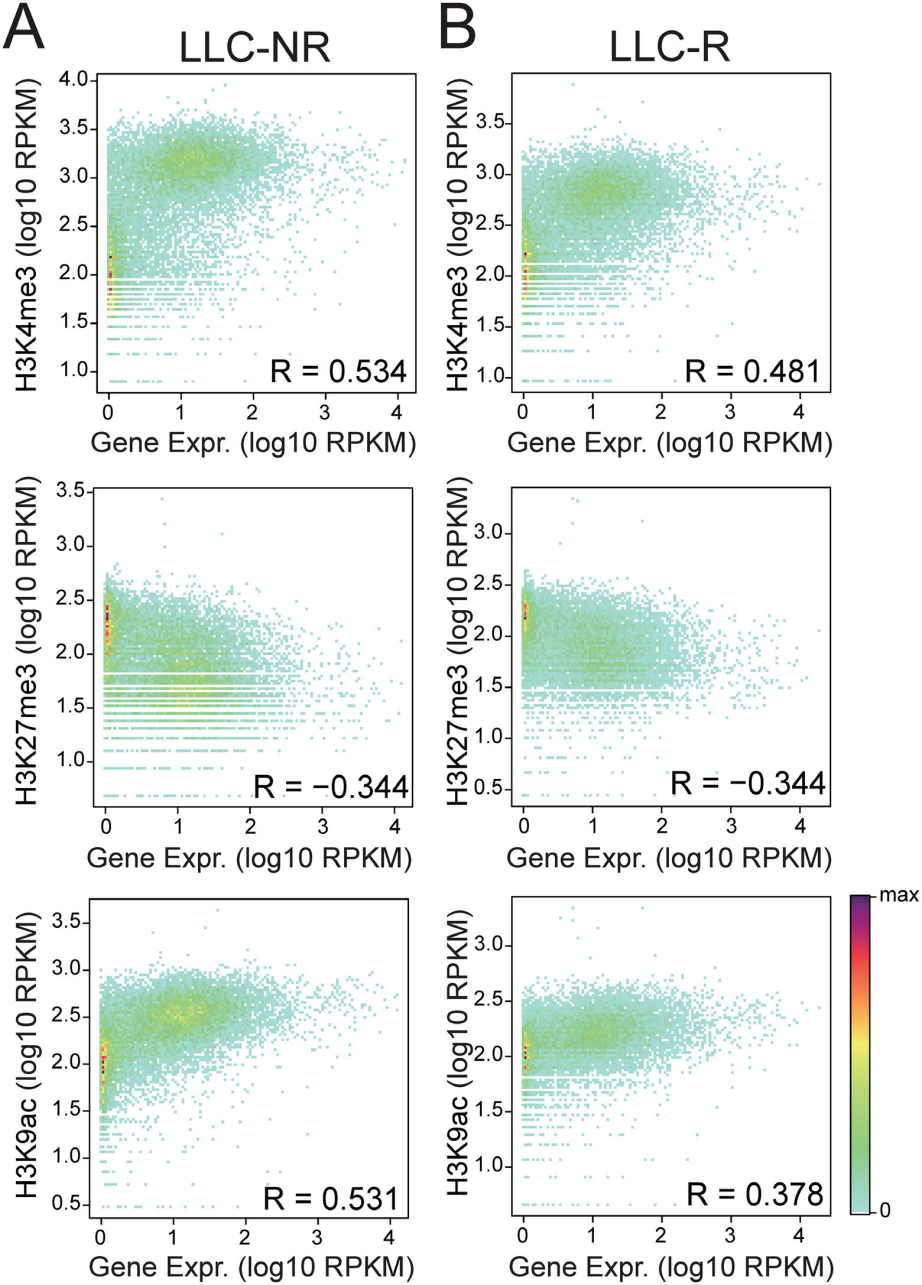
Correlation of histone modification profiles at all promoters and gene expression. The scatter plots show the correlation in LLC-NR sample (A) and LLC-R sample (B), respectively. The Spearman correlation coefficients are shown.

To gain insights into the role of histone modifications in the regulation of gene transcription during tumor resistance to anti-VEGF therapy in LLC tumors, we profiled genome-wide occupancy of key histone modifications (H3K4me3, H3K27me3, H3K36me3, and H3K9ac) for anti-VEGF therapy using high throughput sequencing. It has been shown that deposition of histone modifications in the promoter regions plays an important role in regulating gene transcription[20]. Therefore, we examined the enrichment of H3K4me3, H3K27me3, and H3K9ac at gene promoters and correlated it with gene expression. Our results show that the occupancy level of active histone marks (H3K4me3 and H3K9ac) are positively correlated with gene expression whereas repressive histone mark (H3K27me3) is negatively correlated with gene expression (Fig 1). This is consistent with the conclusion that histone modification levels can serve predictive marks for gene expression [21].

### Active histone modification levels are increased in promoters in LLC-R tumors

**Fig 2:**
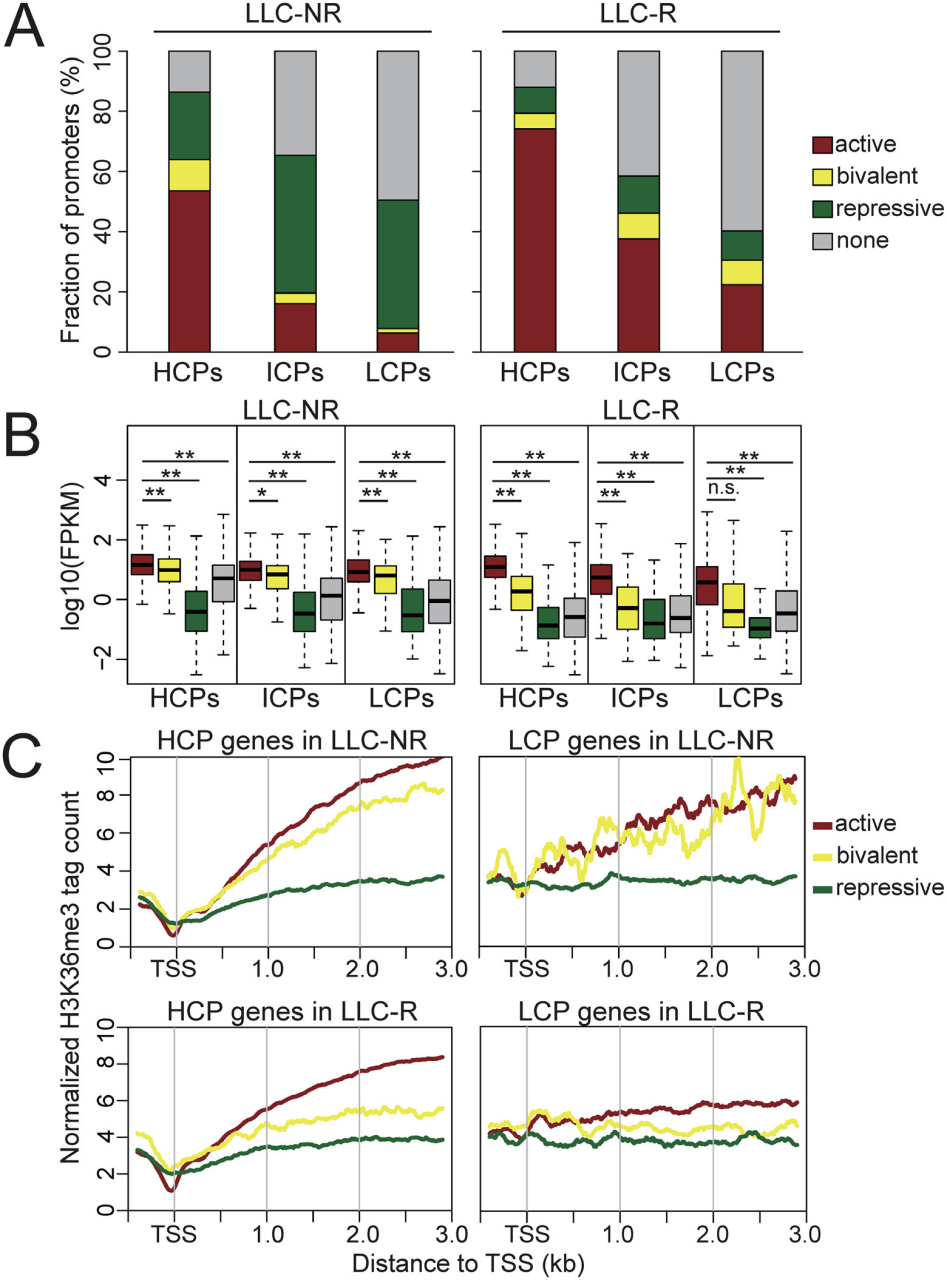
Histone modification levels are predictive for expression of genes categorized by CpG level in promoter. (A) Composition of all promoters classified by histone modification occupancy. “Active” promoters contain occupancy of the active histone marks (H3K4me3 or H3K9ac) higher than the average level of all promoters but occupancy of the repressive histone mark (H3K27me3) lower than the average level. “Repressive” promoters contain occupancy of H3K27me3 higher than the average level and are exclusive of the “active” promoters. “Bivalent” promoters contain occupancy of H3K4me3 or H3K9ac higher than the average level and occupancy of H3K27me3 higher than the average level. “None” promoters are the rest. (B) Box plots show expression levels of genes in each category in (A). (** *p*-value ≤ 0.01, * *p*-value ≤ 0.05, n.s. *p*-value > 0.05, Student’s *t* test.) (C) H3K36me3 profiles in different sets of genes classified in (A). HCP: CpG-rich promoters, ICP: intermediate-CpG-level promoters, LCP CpG-poor promoters.

Motivated by the fact that promoters with different CpG level have distinct histone modification state [19], we further proceeded with analysis of histone modification state in different groups of promoters categorized by CpG level and its correlation with gene expression. We grouped promoters by their CpG level and obtained 9,624 CpG-rich promoters (HCPs), 2,801 intermediate CpG level promoters (ICPs) and 2,543 CpG-poor promoters (LCPs). Active histone modifications (H3K4me3 or H3K9ac) are highest in HCPs, intermediate in ICPs, and lowest in LCPs in both LLC-NR and LLC-R tumors (Fig 2A). Careful examination found that active histone modification levels in each group of promoters are higher in LLC-R than in LLC-NR tumors. Conversely, the repressive histone modification level in all promoters is much lower in LLC-R than in LLC-NR tumors. This suggests that the genes whose promoters with increased active histone modification level may contribute to tumor resistance to anti-VEGF therapy.

Consistent with the predictive role in gene expression of histone modifications, we found that regardless of CpG level in promoters, genes whose promoter with active histone modifications had highest transcription level, genes whose promoter with bivalent histone modifications had second highest transcription level, and genes whose promoter with repressive histone modification had lowest transcription level (Fig 2B). H3K36me3 is a transcription elongation mark and enriched across the gene body [20]. Indeed, H3K36me3 level is positively correlated with gene transcription level. That is, H3K36me3 level is highest in genes whose promoter with active histone modifications, intermediate in genes whose promoter with bivalent histone modifications, and lowest in genes whose promoter with repressive histone modification (Fig 2C).

### Histone modification change in promoters involves in tumor resistance to anti-VEGF therapy

**Fig 3:**
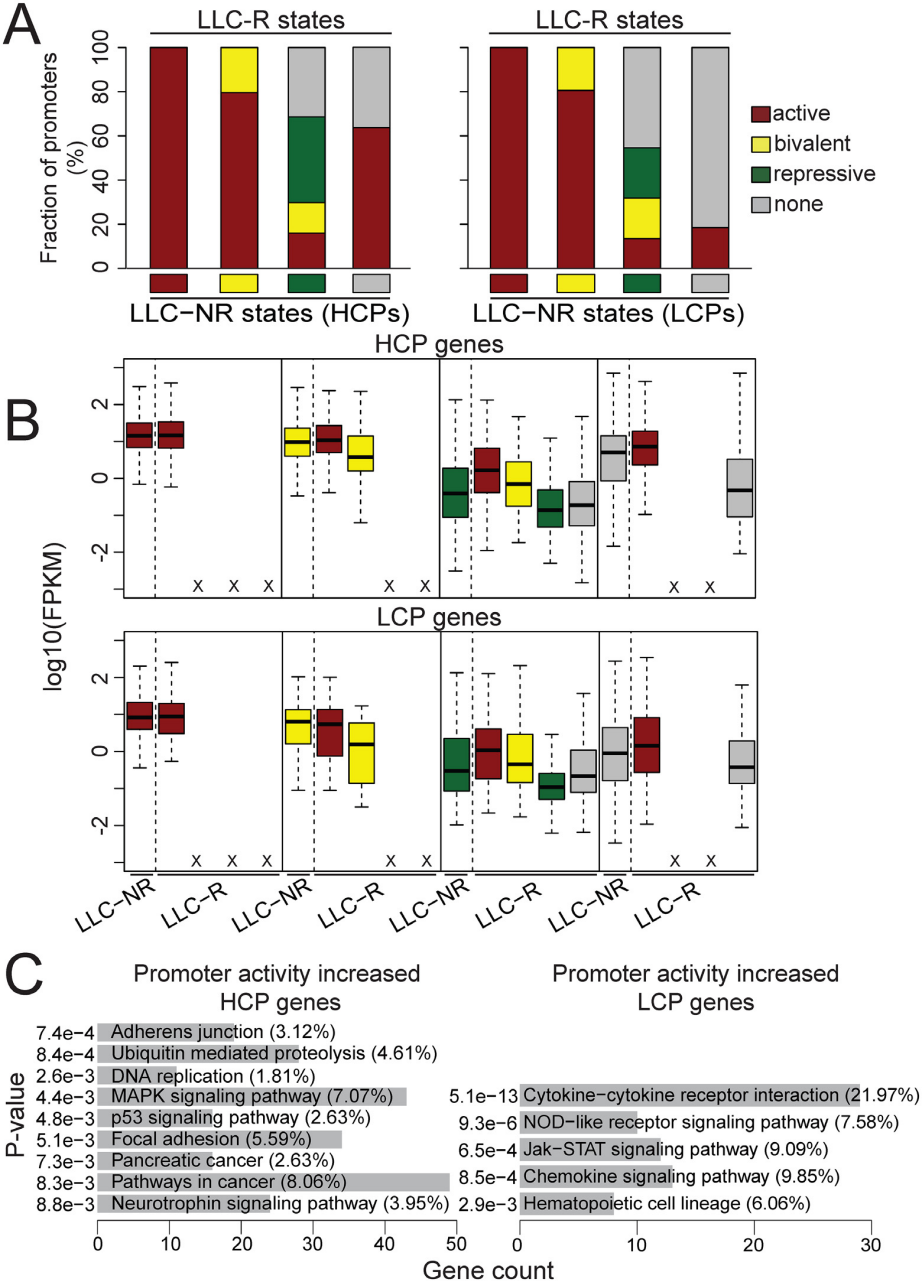
Histone modification state changes in promoters regulate gene expression in tumor resistance. (A) Proportion of all promoters in LLC-NR with a specific histone modification state that change to each histone modification state in LLC-R. Histone modification states in LLC-NR cells are indicated below each bar. The promoter classification is the same as in Fig 2. (B) Expression level changes of each category of genes classified in (A) during tumor resistance. Genes whose promoters gain active or lose repressive histone modifications are associated with increased expression. (C) GO analysis of genes whose promoters gain active or lose repressive histone modifications during tumor resistance. Gene count as a percentage of genes in each GO term is indicated in parentheses.

To investigate how histone modifications change between LLC-NR and LLC-R tumors and its functions in tumor resistance to anti-VEGF therapy, we analyzed the transition of histone modification states in promoters between LLC-NR and LLC-R tumors. Surprisingly, all “active” promoters in LLC-R tumor remain “active” in LLC-NR tumor (Fig 3A). Approximately 80% of “bivalent” promoters in the sensitive tumor cells resolve to the “active” status in the resistant tumor cells. The remaining “bivalent” promoters in LLC-NR tumor remain the “bivalent” status in LLC-R tumor. The pattern of histone modification changes in “active” and “bivalent” promoters is the same between HCPs and LCPs. In contrast, higher percentage of “repressive” LCPs lose H3K27me3 mark from LLC-NR to LLC-R than “repressive” HCPs. Contrarily, more “none” HCPs become “active” from LLC-NR to LLC-R than “none” LCPs (Fig 3A). The impact of the histone modification change in promoters on gene expression is consistent with the predictive roles of histone modification for gene expression. That is, increase of active marks or loss of repressive marks results in upregulation of gene transcription, vice versa. For example, there is no difference in expression of the genes whose promoters remain “active” in both LLC-NR and LLC-R. The genes showed increased expression whose promoter states change from “repressive” or “none” to “active” (Fig 3B). Functional analysis of HCP genes with gain of active histone modifications or loss of repressive histone modifications in their promoter revealed that they were enriched for “MAPK signaling pathway”, “p53 signaling pathway”, “Pathways in cancer” (Fig 3C). The increased activity of genes in MAPK signaling pathway consists with the previous finding that VEGF-C can induce VEGFR2 phosphorylation [22] and thus stimulate angiogenesis [23]. Gene Ontology (GO) analysis of counterpart LCP genes identified enrichment for “Chemokine signaling pathway”, “Hematopoietic cell lineage”, et al. (Fig 3C). This suggests that histone modification changes regulate different sets of genes with distinct functions and exploit alternative signaling pathways to contribute to resistance to anti-angiogenesis therapy.

### Histone modification changes regulate expression of angiogenesis related genes that may contribute to tumor resistance to anti-VEGF therapy

**Fig 4:**
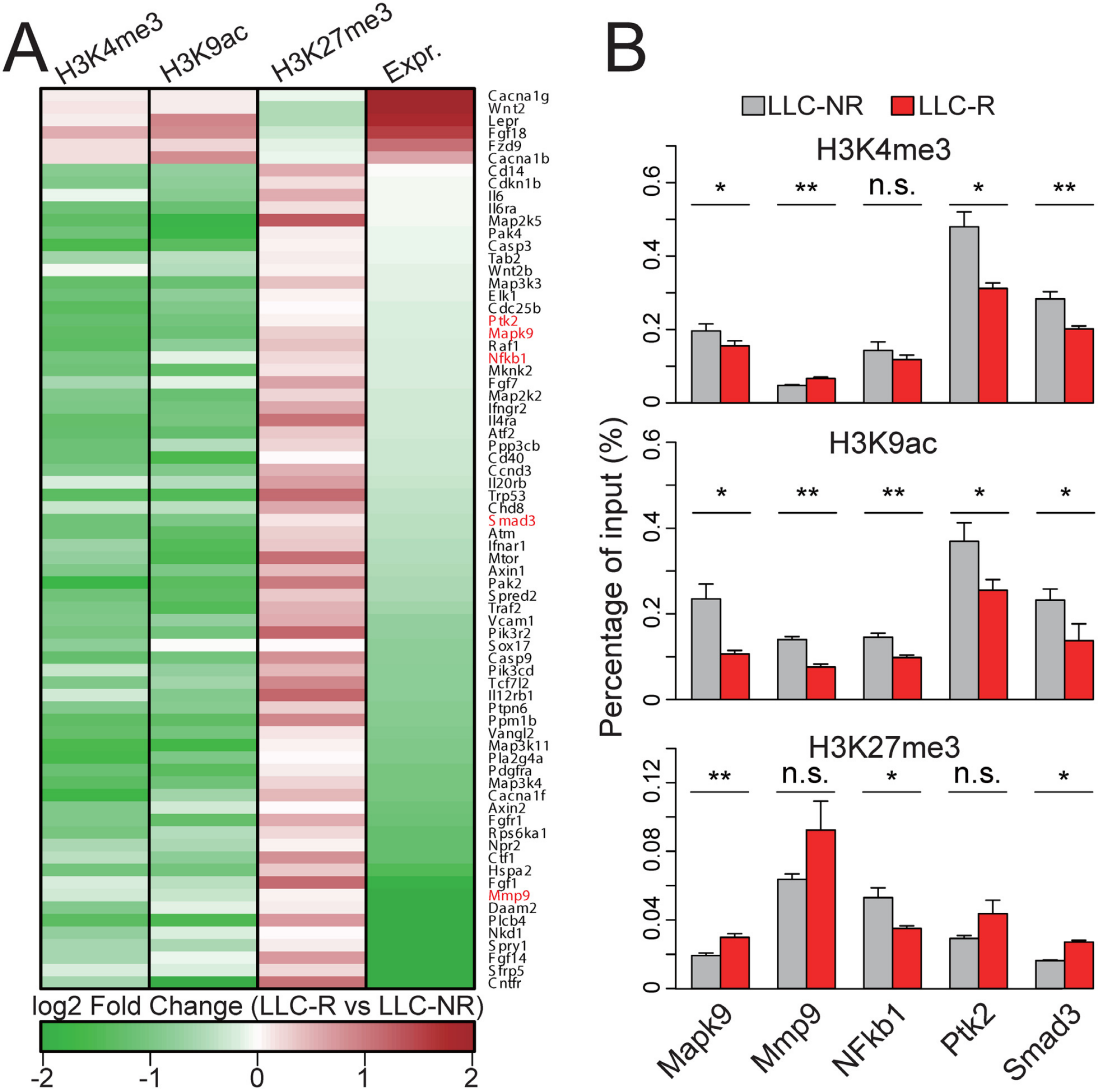
Histone modification state changes correlate with expression of angiogenesis-related genes. (A) Heatmaps show the fold changes of histone modifications in the promoters (-1 kb to +0.5 kb around TSS) of angiogenesis-related genes and their expression fold change. Genes selected for experimental validation are highlighted in red. (B) Quantification of histone modification occupancy in the promoters of selected genes by ChIP-qPCR. (** *p*-value ≤ 0.01, * *p*-value ≤ 0.05, n.s. *p*-value > 0.05, Student’s *t* test.)

Our previous transcriptomic profiling study has shown that differentially expressed genes in tumor resistance are enriched in the biological processes related to angiogenesis and inhibition of VEGF-C in resistant tumor cells can restore sensitivity to anti-VEGF therapy[16]. To investigate the role of histone modifications in tumor resistance, we correlated histone modification changes in angiogenesis related genes with their expression changes. The results show that histone modification changes in the promoters of these genes consist with the expression changes. Specifically, up-regulated genes in resistant tumor cells show increase in active histone modification marks and decrease repressive histone modification mark. Conversely, down-regulated genes show decrease in active histone modification marks and increase repressive histone modification mark (Fig 4A, Table 1). We further selected some key angiogenesis related factors for further validation using quantitative PCR (Fig 4B). For example, Smad3 is a direct target of VEGF signaling, which is involved in epithelial—mesenchymal transition (EMT) [24]. While VEGF itself is a direct target of NF-kB [25] and Mapk9 [26]. Moreover, Mmp9 is a cell surface protein which is responsible for matrix remodeling during angiogenesis and cell invasion [27]. Although Ptk2 has not been proved to interact with VEGF signaling directly, its receptor, Tie2, is required in angiogenesis [28]. Histone modification changes at promoters in most of these selected genes are consistent with the sequencing results.

**Table 1:** Association of gene expression with histone modifications in drug-resistance isograft samples (LLC-R).

Gene pathway	Upregulated in LLC-R	Downregulated in LLC-R
**JAK-STAT pathway**	Lepr	Cntfr,Spry1,Ctf1,Ptpn6,Il12rb1,Pik3cd,Pik3r2,Spred2,Ifnar1,Socs5,Tyk2,Il20rb,Ccnd3,Il4ra,Ifngr2,Il6ra,Il6
**Wnt pathway**	Fzd9,Wnt2	Sfrp5,Nkd1,Plcb4,Daam2,Axin2,Vangl2,Tcf7l2,Sox17,Ctnnbip1,Axin1,Smad3,Chd8,Trp53,Fzd1,Ccnd3,Ppp3cb,Nfatc3,Lrp6,Mapk9,Ruvbl1,Wnt2b,Camk2g,Rac3,Nlk,Rhoa,Fbxw11,Csnk2b,Dvl1
**VEGF pathway**		Npr2,Pla2g4a,Pik3cd,Casp9,Pik3r2,Ppp3cb,Map2k2,Raf1,Ptk2,Rac3
**MAPK pathway**	Cacna1b, Fgf18, Cacna1g	Fgf14,Fgf1,Hspa2,Rps6ka1,Fgfr1,Cacna1f,Map3k4,Pdgfra,Ptpn7,Pla2g4a,Map3k11,Ppm1b,Hspa1b,Bdnf,Gna12,Mecom,Traf2,Pak2,Map4k4,Rasa1,Trp53,Flna,Map4k1,Ppp3cb,Atf2,Rasa2,Nfatc3,Map2k2,Fgf7,Taok2,Mknk2,Nfkb1,Raf1,Mapk9,Dusp10,Cdc25b,Elk1,Map3k3,Arrb2,Tab2,Casp3,Gadd45g,Rac3,Mknk1,Nlk,Map2k5,Cd14,Stk4,Mapk8ip3,Taok1
**ERBB pathway**		Pik3cd,Pik3r2,Pak2,Mtor,Nck2,Map2k2,Raf1,Abl2,Mapk9,Ptk2,Gab1,Elk1,Camk2g,Abl1,Pak4,Cdkn1b,Shc4
**NF-κB pathway**		Vcam1,Birc3,Traf2,Myd88,Btk,Icam1,Lrdd,Atm,Cd40,Xiap,Nfkb1,Ly96,Birc2,Tab2,Ube2i,Cflar,Cd14,Csnk2b
**Other genes related to angiogenesis**		Mmp9,Ifnar1,Zeb2,Smad3,Vim,Cope

Taken together, our results suggest that histone modifications not only serve as predictive marks for gene expression but also contribute tumor resistance to VEGF-therapy through regulating angiogenesis related genes. Our findings shed new insights on the mechanisms underlying tumor resistance to prolonged anti-VEGF treatment and suggest a potential alternative to restore tumor sensitivity through epigenetic regulation.

## Discussion

The epigenetic drivers of tumor drug resistance have been widely recognized in a variety of cancer types. In NSCLC, it is reported that histone demethylases are required during establishment of drug tolerance, while histone deacetylases (HDAC) activity is also related to the development of drug resistance [29]. In another study in NSCLC and neuroblastoma cell lines, Hajji et al. employed HDAC inhibitors to investigate the drug resistance mechanisms and found that both the deacetylase hMOF and its target H4K16ac play key roles in sensitivity of drug resistant calls [30]. Besides, H3K9 methylation is critical to the initial step of lymphomagenesis, therefore its methyltransferase Suv39h1 may be important to lymphomas senesce after drug therapy [31].

Inspired by the previous studies, we explored the role of histone modification in association and regulation of drug resistance using a Lewis lung carcinoma mouse isograft model with ChIP-seq and bioinformatics analyses. It was found that histone modification status was altered in some sets of genes with specific functions during tumor resistance, particularly angiogenesis-related pathway genes. These findings indicate that histone modifications in these genes likely contribute to drug resistance in Lewis lung carcinoma isograft tissues. However, further study is needed to verify expression of angiogenesis genes for association with drug resistance (specially, the VEGF inhibitor-induced resistance in LLC cells) and investigate the underlying molecular mechanisms by which angiogenesis genes lead to drug resistance.

ChIP-seq technique is a method to analyze protein interactions with DNA [32, 33]; thus, to analyze how histone modifications, transcription factors and other chromatin-associated proteins influence phenotype-affecting mechanisms. The present study utilized four different antibodies to recognize histone modifications to analyze how histone modifications regulate gene expression for association with drug-resistance according to a previous study which evaluated genomic maps and comparative analysis of histone modifications in human and mouse [34]. Notably, different histone modifications, such as H3K4me3, H3K27me3, H3K36me3, and H3K9ac have a different effect on gene expression, i.e. H3K4me3 and H3K36me3 associate with initiation and elongation of RNA polymerase II respectively, and both are indicative of actively transcribed genes [35, 36]. H3K9ac is another marker for transcriptionally active chromatin which plays key roles in pluripotency [37]. Whereas, H3K27me3 is a known marker of gene repression [38]. Thus, in the present study, we utilized specific antibodies that can recognize these four different histone modifications to identify genes or gene pathways for association with drug resistance in both drug-sensitive and resistance lung cancer cell isograft tissues. We performed bioinformatics approaches to predict the enriched region and occupancy of each histone modification in whole genome. And further analysis indicated that changed chromatin states affected the promoter activity of angiogenesis related genes.

Previous studies have associated expression of angiogenesis related genes with lung cancer development and progression [39], whereas angiogenesis inhibitors were effective in treatment of advanced NSCLC [40]. Moreover, it was reported that increased VEGFR-2 copy number was associated with chemo-resistance and shorter survival in NSCLC patients who receive adjuvant chemotherapy [41], while another recent study showed that capsaicin, an active component of chili peppers, was able to restrain angiogenesis to achieve more efficient and cogent therapy of resistant NSCLC cells. Capsaicin treatment induced activation of p53-SMAR1 auto-regulatory loop to repress COX-2 expression that restrained HIF-1α nuclear localization, which consequently downregulated VEGF expression to stop endothelial cell migration and network formation, pre-requisites of angiogenesis in tumor micro-environment in NSCLC cells [42]. These results revealed that drug-resistant lung cancer isograft tissue expressed high levels of angiogenesis-related genes. Moreover, some other pathways were also identified in the present study, such as the MAPK, p53, Wnt, JAK-STAT, ERBB, NF-κB, and ubiquitin mediated proteolysis signaling pathways, which altered during the process of drug-resistance of murine lung cancer isografts *in vivo*.

However, the present study is just proof-of-principle and there are some limitations; for example, the drug-resistant LLC cell subline was generated by treatment of parental LLC cells with a potent VEGF inhibitor, VEGF-Trap, through repeated in vivo selections, which is mostly limited to angiogenesis-related gene alterations. Although other gene pathways identified simultaneously, the conclusion may not be general to other drug-resistance. Moreover, the present study only limitedly verified the ChIP-seq data using qPCR and there is a big room for further in-depth analysis. In summary, the present study identified and evaluated the role of histone modifications in regulation of VEGF inhibitor-resistance of LLC cells. Future study will be designed to investigate the underlying molecular mechanisms by which expression of angiogenesis genes lead to VEGF inhibitor-induced drug resistance.

## Supporting Information

S1 Table Statistics of read mapping results. (DOC)

S2 Table Primers used for ChIP-qPCR. (DOC)
