# Supplementary material for: Profiling Analysis of Histone Modifications and Gene Expression in Lewis Lung Carcinoma Murine Cells Resistant to Anti-VEGF Treatment
Source: PLoS One. 2016 Jun 30;11(6):e0158214. doi: 10.1371/journal.pone.0158214 (PMC4928805; doi:10.1371/journal.pone.0158214)
Supplement: S1 Table — (DOC) [file pone.0158214.s001.doc]

**S1 Table. Statistics of read mapping results.**

| **ChIP data** | **Samples** | **Total Read** | **Uniquely mapped** | **Percentage of Uniquely mapped (%)** |
| --- | --- | --- | --- | --- |
| **H3K4me3** | LLC-NR | 24,894,974 | 14,647,219 | 58.84 |
|  | LLC-R | 22,406,188 | 12,127,176 | 54.12 |
| **H3K9ac** | LLC-NR | 58,276,318 | 48,114,878 | 82.56 |
|  | LLC-R | 36,846,363 | 27,556,122 | 74.79 |
| **H3K27me3** | LLC-NR | 32,560,362 | 25,509,157 | 78.34 |
|  | LLC-R | 76,191,384 | 54,675,851 | 71.76 |
| **H3K36me3** | LLC-NR | 25,493,484 | 18,638,183 | 73.11 |
|  | LLC-R | 62,024,358 | 43,248,700 | 69.73 |

LLC-NR, drug-sensitive isograft samples; LLC-R, drug-resistant isograft samples.

**S2 Table. Primers used for ChIP-qPCR.**

| **Gene** | **Forward primer** | **Reverse primer** |
| --- | --- | --- |
| **Smad3** | 5'-CTCCTCCTCCTCCTCCTCTG-3' | 5'-TGCGTGAAACGTAGACTTGG-3' |
| **Ptk2** | 5'-AGATCCTTAGCCGCACACAC-3' | 5'-ACAGAAAGCCCATGAACCTC-3' |
| **Nfkb1** | 5'-GCGAAACCTCCTCTTCCTG-3' | 5'-AAGTGAGAGAGTGAGCGAGAGAG-3' |
| **Mapk9** | 5'-ACACTGTAGTCGCAAAGTCACG-3' | 5'-TGCACAGAGTTAGGGATCCAG-3' |
| **Mmp9** | 5'-AGGATGAAGCTTCTGCTTGC-3' | 5'-CTTGGGGAAGGAAAGATGAG-3' |
